# Effect and Process Evaluation of e-Powered Parents, a Web-Based Support Program for Parents of Children With a Chronic Kidney Disease: Feasibility Randomized Controlled Trial

**DOI:** 10.2196/jmir.9547

**Published:** 2018-08-01

**Authors:** Wytske W Geense, Betsie GI van Gaal, Jaqueline L Knoll, Nienke M Maas, Gerjo Kok, Elisabeth AM Cornelissen, Maria WG Nijhuis-van der Sanden

**Affiliations:** ^1^ IQ Healthcare Radboud Institute for Health Science Radboud University Medical Center Nijmegen Netherlands; ^2^ Institute of Nursing HAN University of Applied Science Nijmegen Netherlands; ^3^ Department of Pediatric Nephrology Amalia Children’s Hospital Radboud University Medical Center Nijmegen Netherlands; ^4^ Department of Medical Psychology Radboud University Medical Center Nijmegen Netherlands; ^5^ Department of Work & Social Psychology Maastricht University Maastricht Netherlands

**Keywords:** child, chronic kidney failure, chronic kidney disease, effect evaluation, health promotion, process evaluation

## Abstract

**Background:**

Parents of children with chronic kidney disease (CKD) experience high levels of stress in the daily management of their child’s illness. Parents need continuously available support and information, yet online support programs are lacking. e-Powered Parents was developed to fill this gap; it is an online program consisting of (1) medical information, (2) an interactive part, and (3) four training modules (stress management, setting limits, communication, and coping). Prior to a large-scale evaluation, we conducted a feasibility study that consisted of an effect study and a process evaluation.

**Objective:**

The objectives of our study were to (1) identify the outcome measures that are most likely to capture the potential benefit, (2) evaluate the potential effectiveness and effect size, and (3) evaluate recruitment, reach, the dose received, and context.

**Methods:**

We conducted a feasibility study with a two-armed, wait-list randomized controlled trial (RCT). Prior to baseline, parents (n=146) were randomly allocated to group 1 or group 2. After completing the baseline questionnaire, parents in group 1 were given access to e-Powered Parents, while those in group 2 received usual care. At the 6-month follow-up (T1), all parents received a questionnaire and parents in group 2 were given access to e-Powered Parents as well. After 1.5 years, through an extra measurement (T2), we evaluated the effect of long-term exposure. Outcomes were the child’s quality of life (Child Vulnerability Scale), parental stress (Pediatric Inventory for Parents) and fatigue (Multidimensional Fatigue Inventory), self-efficacy in communication with health care professionals (Perceived Efficacy in Patient-Physician Interactions, PEPPI-5), and parental perceptions of family management (Family Management Measure). Floor and ceiling effects and percentage of parents showing no change in scores were calculated. We used linear mixed models to evaluate the potential effectiveness and effect sizes using the intention-to-treat and per-protocol analyses. In the process evaluation, we evaluated recruitment, reach, the dose received, and context using a questionnaire sent to the parents, log-in data, and a focus group interview with health care professionals.

**Results:**

At T1 (n=86) and T2 (n=51), no significant effects were found on any of the five outcomes. The PEPPI-5 showed ceiling effects and high percentages of parents showing no change between the measurement times. The information and interactive part of the intervention were used by 84% (57/68) of the parents in group 1 and 49% (32/65) of the parents in group 2. The information pages were visited most often. Overall, 64% (85/133) of the parents logged in to the training platform and 31% (26/85) actually used the training modules.

**Conclusions:**

We did not observe any significant effect on any of the outcomes. This could possibly be explained by the minimal use of the intervention and by parents’ heterogeneity. For continued participation, we recommend a tailored intervention and further studies to find out whether and how online programs could be used to support parents in the management of their child’s CKD.

**Trial Registration:**

Netherlands Trial Registry NTR4808; http://www.trialregister.nl/trialreg/admin/rctview.asp?TC=4808 (Archived by WebCite at http://www.webcitation.org/719rCicvW)

## Introduction

Parents play a key role in the management of their child’s illness. However, parents of children with chronic kidney disease (CKD) experience high levels of stress. Complications such as infections, bone diseases, poor growth and development, and kidney failure are frequently seen among such children [1,2]. Mortality among children with CKD remains 30 times higher than that among healthy children, despite renal replacement therapy or kidney transplantation [2]. Moreover, care for these children is complex due to complicated medication schedules, nutritional restrictions, and procedures such as hemodialysis or peritoneal dialysis [2]. It is, therefore, not unusual that parents experience difficulties in balancing the needs of their sick child with their own responsibilities, such as other children, family members, work, and social life [3]. Parents with significant emotional distress of their own and poor family function can negatively affect their child’s health outcomes as well as their quality of life [4]. Supporting these parents is, therefore, necessary to help them cope with the difficulties encountered in all the stages of their child’s CKD.

In recent years, more attention has been paid to the development of psychoeducational support programs for parents of children with chronic diseases to assist families with the day-to-day management of their child’s chronic disease and its consequences [3,5]. Support programs for parents can take many forms, consisting, for example, of a simple provision of information via written materials, computer programs, internet programs, and group interventions. Eccleston et al [5] concluded in their Cochrane review that more psychological interventions are needed that directly target the parents of children with chronic illness. In 2008, Swallow et al [6] described how parents of children with CKD needed continuously available, accessible, and reliable support. Although in recent years, an increasing number of online support programs have been developed for parents of children with chronic diseases, such as diabetes mellitus [7,8], cystic fibrosis [9,10], and asthma [11], online support programs for parents of children with CKD are as yet lacking.

To fill this gap, we developed *e-Powered Parents (Mijn Kinderniernet* in Dutch), an online support program for parents of children with CKD, using intervention mapping. Intervention mapping is a protocol for the systematic development of theory- and evidence-based health promotion interventions, consisting of six different steps [12]. The completion of all these steps serves as a blueprint for designing, implementing, and evaluating an intervention based on the foundation of theoretical, empirical, and practical information [12]. After conducting a needs assessment with parents (consisting of 5 focus group interviews) [13] and with health care professionals (step 1), defining program objectives (step 2), and searching for theories and selecting practical applications (step 3), we developed *e-Powered Parents* (step 4; Multimedia Appendix 1). Subsequently, a plan was designed for the adoption, implementation, and sustainability of *e-Powered Parents* (step 5) [14].

The last step of intervention mapping (step 6) includes the planning for evaluation [15]. We decided to conduct a feasibility randomized controlled trial (RCT) because prior to a large-scale evaluation of developing and testing interventions, feasibility studies are essential [16]. The aims of this feasibility study were to identify outcome measures that are most likely to capture the potential benefit and to evaluate the potential effectiveness and effect size of the program. We also conducted a process evaluation to understand the results of the effect evaluation. According to the Medical Research Council [17], process evaluations are an essential part of designing and testing complex interventions to look inside the so-called “black box” to see what happened in the program and how that could affect program outcomes [18].

## Methods

### Objectives

This feasibility study consisted of an effect and process evaluation that covered 3 objectives. The objectives of the effect evaluation were (1) to identify outcome measures most likely to capture the potential benefit and (2) to evaluate the potential effectiveness and effect size.

The objective of the process evaluation was (3) to evaluate the recruitment, reach, dose received, and context of *e-Powered Parents*.

### Study Design and Randomization

For the effect evaluation, we conducted a feasibility, two-armed RCT at the Pediatric Nephrology Unit of a single university medical center in the Netherlands. In the course of the study, we changed the design to a wait-list RCT (as described below). For the process evaluation, we conducted a quantitative and qualitative study alongside the RCT.

**Figure 1:**
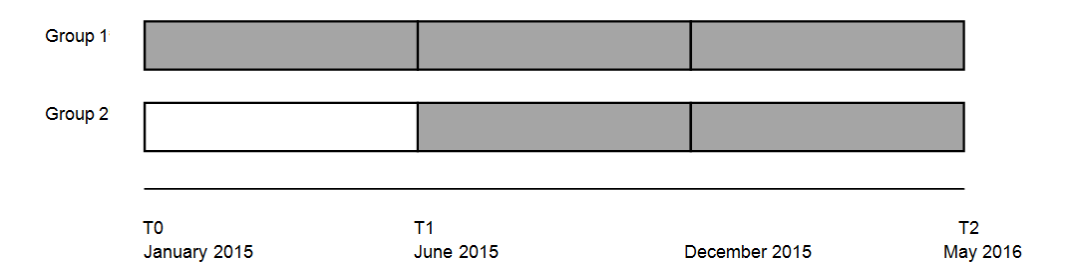
Study design. From June 2015 until December, e-Powered Parents was improved. Group 1: access to e-Powered Parents between T0-T2; Group 2: access to e-Powered Parents between T1-T2.

Randomization, stratified on CKD stages at the family level (see subsection Participants and Recruitment) and performed blind by a statistician using a computer random number generator, was used to allocate equal numbers of parents to the intervention group (group 1) and control group (group 2). After the baseline survey (T0) in January 2015, parents in group 1 were given access to the intervention *e-Powered Parents*, while parents in group 2 received the usual care (Figure 1). After the first follow-up measurement (T1) in June 2015, the Pediatric Nephrology Unit implemented *e-Powered Parents* as part of daily care for children with CKD. Subsequently, parents in group 2 were given access to *e-Powered Parents* as well. We used the feedback from the parents in group 1 at T1 to improve *e-Powered Parents* for a period of 5 months (July to November 2015), adding more information about kidney diseases and supporting videos. After 6 months (May 2016), we conducted an extra measurement (T2) to measure the effect of a longer exposure to the improved program. The design of the study changed thereby from a two-armed RCT to a two-armed, wait-list RCT (Figure 1).

### Ethical Considerations

The Medical Ethics Review Committee of the district Arnhem-Nijmegen approved the study (Registration number 2014/302). All parents received written information about the study’s content and aim and were only included after providing written informed consent. For the extension of the study, the Medical Ethics Review Committee of the district Arnhem-Nijmegen approved the study once more. This trial was registered under the Dutch Trial registration (NTR4808).

### Participants and Recruitment

In this study, Dutch-speaking parents of children aged 0-18 years in 5 different CKD groups were eligible; we included parents of children (1) with hereditary kidney disease (CKD stage I); (2) with nephrotic syndrome (CKD stage I); (3) with chronic kidney failure (CKD stage II-IV); (4) using dialysis (CKD stage V); and (5) with renal transplantation. Both parents of each child were invited to participate. Parents were excluded when their child was not living at home anymore.

Parents were recruited between September and December 2014. They received an information letter, which included an informed consent form. After 3 weeks, reminders were sent and phone calls made, while health care professionals (JK and EC) informed the parents about the study during consultation in the outpatient clinic.

### Sample Size

The first objective of this study was to identify the potential outcome measures; for that reason, a formal power calculation for a test comparing the treatment groups was not appropriate. Our aim was to include as many parents as possible.

### Intervention and Standard Care

*e-Powered Parents* consisted of two different components:

An online community consisting of an informative part comprising information and videos about various kidney diseases, treatment possibilities, diets, and (financial) regulations and an interactive part comprising a forum, chat room, and option to send private messages to share parents’ experiences with other parents and health care professionals.

A Web-based training platform consisting of 4 different training modules (stress management, setting limits, communication, and coping; Multimedia Appendix 1).

Parents in both groups 1 and 2 received standard care.

### Data Collection and Measurements

See Table 1 for data collection regarding the effect evaluation and process evaluation. We collected data after randomization (T0), 6 months (T1), and 1.5 years (T2) through a Web-based questionnaire. Additional data were collected by extracting the log-in data of *e-Powered Parents* as well as through a focus group interview.

#### Effect Evaluation

We selected five potential main outcomes to identify the outcome measures that were most likely to capture the potential benefit (first objective) and to evaluate the potential effectiveness and effect size (second objective):

1. *Child’s quality of life* was measured using the validated Dutch version [19] of the Child Vulnerability Scale (CVS) [20], a proxy instrument measuring the parental perceptions of a child’s vulnerability, which is related to the child’s health-related quality of life [21]. Each of the 8 items is rated on a 4-point Likert scale (0=“definitely true”; 3=“definitely false”). The total score ranges from 0 to 24. Higher scores indicate an increased vulnerability.
2. *Pediatric-related parental stress* was measured using the validated Dutch version [22] of the Pediatric Inventory for Parents (PIP) [23], a 42-item questionnaire covering 4 domain scales: “Communication,” “Emotional distress,” “Medical care,” and “Role function.” Each item is rated on a 5-point Likert scale (1=“not at all”; 5=“extremely”). The 4 domain scores are summed up, resulting in a total overall frequency score and a total difficulty score. Higher scores indicate a higher frequency and difficulty.
3. *Parental fatigue* was measured using the validated Dutch version of the Multidimensional Fatigue Inventory (MFI) [24], a 20-item instrument covering 5 dimensions: “General fatigue,” “Physical fatigue,” “Mental fatigue,” “Reduced motivation,” and “Reduced activity.” Each item is rated on a 5-point Likert scale, ranging from “Yes, that is true” to “No, that is not true.” Higher scores indicate a higher degree of fatigue.
4. *Self-efficacy in the communication with health care professionals* was measured using the validated Dutch version [25] of the Perceived Efficacy in Patient-Physician Interactions (PEPPI-5) [26]. Participants rate each of the 5 items on a 5-point Likert scale, with 1 representing “Not at all confident” and 5 representing “Very confident.” Total scores are summed up, ranging from 5 to 25; higher scores indicate a higher perceived self-efficacy in patient-physician interactions.
5. *Parental perceptions of the family management of chronic conditions* were measured using the Family Management Measure (FaMM) [27]. The FaMM measures how families manage caring for a child with a chronic condition and the extent to which they incorporate condition management into their everyday family life. The FaMM consists of 45 items, covering 5 dimensions: “Child’s daily life,” “Condition management ability,” “Condition management effort,” “Family life difficulty,” and “View on condition impact.” The sixth dimension, “Parental mutuality” (8 items) is additional for partnered parents. Higher scores on the scale “Child’s daily life,” “Condition management ability,” and “Parental mutuality” indicate a greater ease in managing the child’s condition. Higher scores on the other three scales indicate a greater difficulty in managing the condition. Because no validated translation was available for the FaMM, we decided to translate it using forward translation and expert panel back-translation by translators and the members of our team.

#### Process Evaluation

For the process evaluation (the third objective), we used 4 out of 6 components of the model of Linnan and Steckler [28]: (1) “recruitment,” (2) “reach,” (3) “the dose received,” and (4) “context.” The components “dose delivered” and “fidelity” were not relevant for the Web-based intervention *e-Powered Parents*. We used quantitative research methods for the components “recruitment,” “reach,” and “the dose received” and a qualitative research method for the component “context.”

- *Recruitment* consists of the procedures used to approach, attract, and maintain parents; the number of parents who agreed to participate in the study; and the experienced barriers and reasons for nonparticipation [15,18,28]. We used Excel to register parents who wanted to participate. Furthermore, we added open-ended questions to the Web-based survey at T1 and T2 in order to gain insights into the experienced barriers and reasons for nonparticipation.
- *Reach* is the proportion of parents who actually visited *e-Powered Parents* [28]. In Excel, we registered the parents who had logged in at the community (informative and interactive part) or training platform and had set up an account.
- *The dose received* could be divided into exposure and satisfaction. Exposure is the extent to which the parents actively engaged and interacted with *e-Powered Parents* [15,28]. Satisfaction registers the parents’ satisfaction with *e-Powered Parents* [18]. Both the community and the training platform allowed us to extract log-in data, which were used to gain insights into exposure. However, the content and extraction method differed: the log-in data regarding the community included the data of the pages most often visited, number and timing of site visits, page views, and, for example, user device type. We collected these data based on the internet protocol (IP) address in Google Analytics. The log-in data of the training platform included data regarding frequent visits as well as use of the training modules and sessions. Users’ data were registered in the program itself based on the email address and could be extracted using Excel. Furthermore, log-in data for the two websites were collected between T0 and T2. To evaluate parents’ satisfaction, we added open-ended and Likert scale questions regarding the program in general (such as log-in, navigation on the site, and layout) and the relevance and added value to the Web-based survey at T1 (for group 1) and T2 (for groups 1 and 2).
- *Context* includes aspects of the physical, social, and political environment that may affect the implementation of *e-Powered Parents* [15,28]. To explore the context, we conducted a focus group interview with health care professionals of the Pediatric Nephrology Unit after T2. Pediatric nephrologists, (specialist) nurses, social workers, psychologists, and educational workers were invited to participate. We used purposive sampling to ensure that professionals with and without *e-Powered Parents* experience participated in this study. An experienced external moderator posed open-ended questions about the experiences with and implementation of *e-Powered Parents* and possible improvements for the future. One researcher (WG) acted as an observer in the focus group. The focus group interview took approximately 1.5 hours, was audiotaped, and transcribed verbatim.

### Data Analysis

#### Effect Evaluation

To identify the outcome measures most likely to capture the potential benefit (Objective 1), we calculated the percentages of parents scoring zero (floor effect) or full marks (ceiling effects) on the five outcome measures—CVS, PIP, MFI, PEPPI-5, and FaMM. In this calculation, we considered floor and ceiling effects exceeding 20% to be significant [29]. Additionally, we calculated the percentage of parents showing no change in the score between T0, T1, and T2 [30].

**Table 1:** Overview of the measurement methods, objectives, and time points.

Research method and specification					Participants		Outcome (time and group)	
**Quantitative research method**								
	**Effect evaluation (objectives 1 and 2)**							
		**Web-based survey**						
			CVS^a^			Parents		Child’s quality of life (T0-T2: groups 1 and 2)
			PIP^b^			Parents		Parental stress (T0-T2: groups 1 and 2)
			MFI^c^			Parents		Parental fatigue (T0-T2: groups 1 and 2)
			PEPPI-5^d^			Parents		Self-efficacy in communication (T0-T2: groups 1 and 2)
			FaMM^e^			Parents		Family management (T0-T2: groups 1 and 2)
	**Process evaluation (objective 3)**							
		**Log-in data of website**						
			Community^f^			Parents		*The dose received_Exposure*: Pages visited most often (T0-T1: group 1; T1-T2: groups 1 and 2) Number and time of site visits (T0-T1: group 1; T1-T2: groups 1 and 2) Page views (T0-T1: group 1; T1-T2: groups 1 and 2) Time spent on the site (T0-T1: group 1; T1-T2: groups 1 and 2) User device type (T0-T1: group 1; T1-T2: groups 1 and 2)
			Training platform^g^			Parents		*The dose received_Exposure*: Frequency and use of the platform (T0-T1: group 1; T1-T2: groups 1 and 2) Use of training modules and sessions (T0-T1: group 1; T1-T2: groups 1 and 2)
		**Web-based survey**						
			Open questions and 4-point Likert scale^h^			Parents		*Recruitment*: Experienced barriers and facilitators (T0-T1: group 1; T1-T2: groups 1 and 2) *The dose received_Satisfaction*: Parents’ experiences and satisfaction regarding the components of *e-Powered Parent*^i^ (T0-T1: group 1; T1-T2: groups 1 and 2)
		**Own records**			Parents		*Recruitment*: Procedures used to approach and maintain parents (T0-T2: groups 1 and 2) Number of parents who participate (T0-T2: groups 1 and 2) *Reach*: Parents who actually visited *e-Powered Parents* (T0-T1: groups 1; T1-T2: groups 1 and 2)	
**Qualitative research method**								
	**Process evaluation (objective 3)**							
		**Focus group**						
			Open questions^j^			Health care professionals		*Context*: Health care professionals’ experiences and satisfaction with *e-Powered Parents* program components, use, implementation, and how these components could be improved (after T2)

^a^CVS: Child Vulnerability Scale.

^b^PIP: Pediatric Inventory for Parents.

^c^MFI: Multidimensional Fatigue Inventory.

^d^PEPPI-5: Perceived Efficacy in Patient-Physician Interactions.

^e^FaMM: Family Management Measure.

^f^Informative and interaction part of e-Powered Parents (based on IP address of parents).

^g^Based on email address of parents.

^h^Ranging from "totally disagree" to "totally agree."

^i^Information, newsletter, blog, chat, forum, and training modules.

^j^Asked by a moderator during the focus group.

To evaluate the potential effectiveness and effect size (Objective 2), we used linear mixed models with time of measurement (T1 and T2) and exposure to *e-Powered Parents* (at T0, no exposure; at T1, only parents in group 1 exposed, and at T2, parents in both groups exposed) as fixed variables. Three outcome measures (CVS, PIP, and FaMM) were child specific: participating families that consisted of 2 children with CKD had to fill in these questions twice (for every child each). For the analysis of these three outcomes, we took random child effects into account for potential correlation. For nonchild-specific questionnaires (PEPPI-5 and MFI), we took random family effects into account for potential correlation because both partners participated in the study. Also, we conducted intention-to-treat analysis, followed by per-protocol analysis. Results were considered significant if *P*<.05.

We calculated the standardized effect sizes (Cohen *d*) by dividing the mean difference in the change score between groups 1 and 2 by SD at the baseline. Effect sizes>0.8 were considered as large, between 0.5 and 0.8 as medium, between 0.2 and 0.5 as small, and <0.2 as very small.

#### Process Evaluation

In this section, we have described the data analysis for the components *recruitment, the dose received*, and *context*.

##### Recruitment

We analyzed the open-ended questions in the questionnaire using thematic analysis in Atlas.ti, a software program used to analyze qualitative data. Experiences of parents were divided into the program components (information, blog, chat, forum, training modules, etc) and subdivided into positive or negative experiences.

##### The Dose Received

For the community (informative and interactive part), we selected time periods in Google Analytics (eg, January-June 2015) to track the amount of (returning) visitors as well as the information most often read, newsletters, forum topics, chat messages, and private messages sent.

The training platform consisted of 4 modules (stress management, setting limits, communication and coping), with each module consisting of several sessions (Multimedia Appendix 1). Per session, we counted the number of parents finishing that particular session.

For the analysis of the log-in data of the community and training platform, we consulted experts on how best to analyze the data. The 4-point Likert scale questions in the Web-based survey were analyzed in SPSS and merged into 2 categories (Disagree and Agree). Furthermore, frequencies of disagree and agree were counted.

##### Context

Two researchers independently analyzed the focus group interview with the health care professionals using thematic analysis in Atlas.ti. Themes regarding the components of the program (such as “information,” “communication parents with parents,” “communication parents with health care professionals,” “training platform,” and “layout”) and implementation were labeled while within every theme, we defined negative and positive experiences. The health care professionals who participated in the focus group interview received the conclusions of the analysis to check its authenticity.

## Results

### Study Population

We assessed a total of 201 families, whose children were under treatment at the Pediatric Nephrology Unit. Regarding eligibility for the study, 22 families were excluded because of not meeting the inclusion criteria and 9 families for other reasons, such as incorrect personal data. The remaining 170 families were asked to participate, of which 81 families declined either due to lack of time or because they did not feel a need for support.

**Figure 2:**
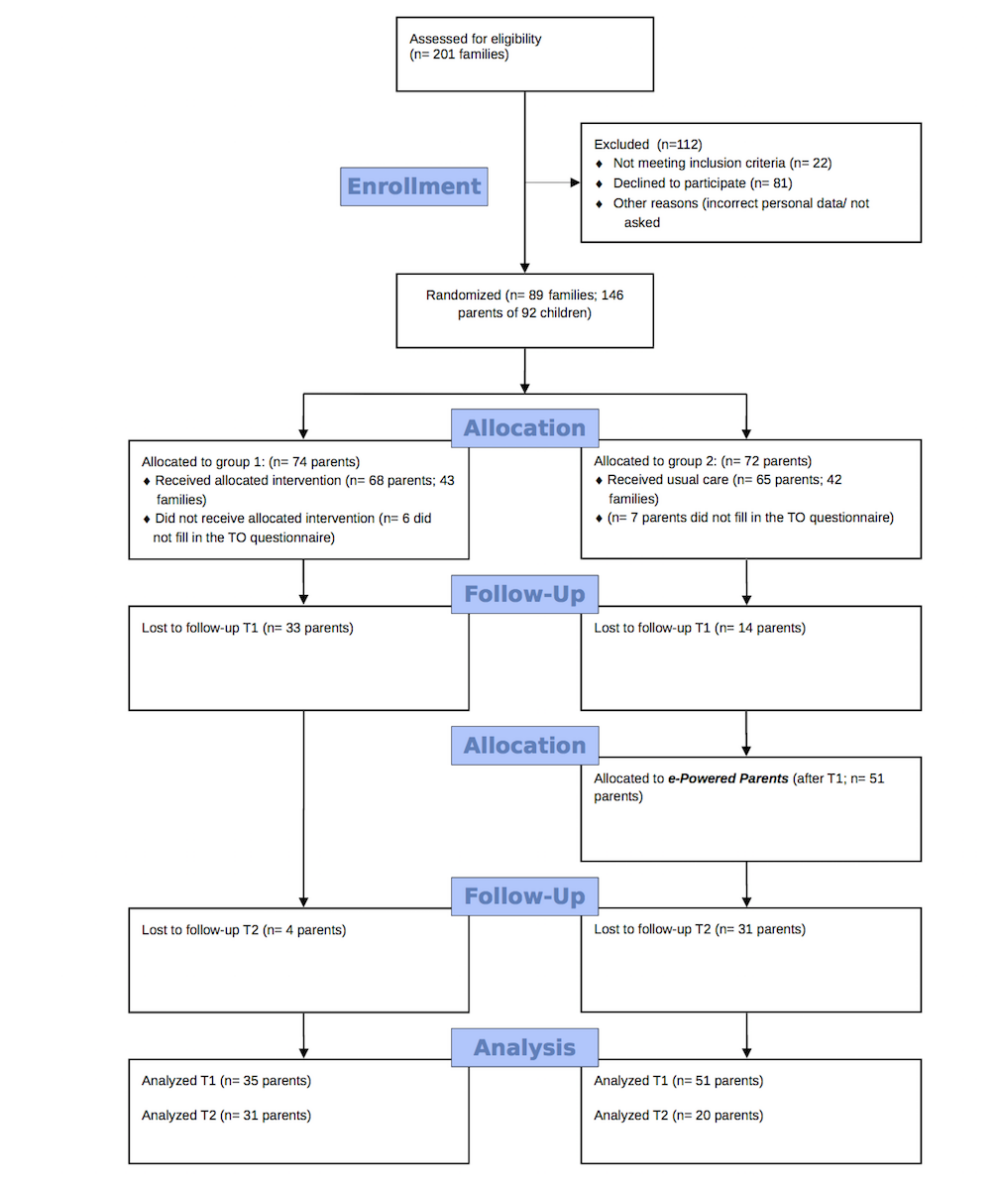
Flow diagram.

Finally, 146 parents of 89 families who were willing to participate were randomized into group 1 (n=74) and group 2 (n=72). After randomization, 13 parents did not fill in the baseline questionnaire, resulting in a total of 133 parents: 68 parents (43 families) in group 1 and 65 parents (42 families) in group 2 (Figure 2).

### Characteristics of Parents and Children

The characteristics of parents in groups 1 and 2 demonstrated similar proportions regarding gender, educational level, and marital status. Regarding children’s characteristics, group 1 had slightly greater proportion of girls and a higher age of children. Overall, the majority of participating parents in both groups were females (83/133, 62%), highly educated (67/133, 50%), married (94/133, 71%), and employed (116/133, 87%). Three families had 2 children with CKD under treatment at the university medical center (Table 2).

### Characteristics of Health Care Professionals

We invited 9 health care professionals to take part in the focus group interview. Of them, 5 eventually participated: 1 nurse practitioner, 1 nurse, 2 pediatric nephrologists, and 1 educational worker. All participants were females.

### Effect Evaluation

When we assessed the percentage of parents scoring 0 (floor) or full marks (ceiling effects) on the five outcome measures, we noticed significant (>20%) ceiling effects at the PEPPI-5 among parents in group 2—T0 (14/65, 21.5%), T1 (14/51, 27.5%), and T2 (5/20, 25%; Multimedia Appendix 2).

The percentage of parents showing no change was high on the PEPPI-5 as well, with 40.6% (56/138) between T0-T1 and 25.4% (35/138) between T0-T2 (Multimedia Appendix 3).

The standardized effect sizes (Cohen *d*) for the five outcome measures were small, ranging in the intention-to-treat analysis from −0.22 to 0.15 and from −0.21 to 0.12 for T0-T1 and T0-T2, respectively (Multimedia Appendix 4). In the per-protocol analysis, these ranged from −0.23 to 0.15 and from −0.20 to 0.12 for T0-T1 and T0-T2 (Multimedia Appendix 5).

In Table 3, we have provided an overview of the mean score on the five outcome measures during T0, T1, and T2. Adjusting for the period of using *e-Powered Parents* (intention-to-treat), the actual use (did parents log in at *e-Powered Parents*: per-protocol), and the experienced stress levels of the parents, no statistically significant differences were found regarding any of these outcomes between parents in groups 1 and 2 between T0-T1 and T0-T2 (Multimedia Appendices 4 and 5).

**Table 2:** Characteristics of parents and their children at the baseline.

Characteristic			Group 1		Group 2
**Parents**			**N=68**		**N=65**
	**Gender, n (%)**				
		Female	42 (62)		41 (63)
		Male	26 (38)		24 (37)
	Age in years, mean (SD)		45.2 (6.5)		42.6 (7.3)
	**Educational level, n (%)^a^**				
		Low	5 (7)		7 (11)
		Medium	27 (40)		26 (41)
		High	36 (53)		31 (48)
	**Marital status, n (%)**				
		Single	12 (18)		11 (17)
		Married	46 (68)		48 (74)
		Divorced	1 (1)		4 (6)
		Widow	1 (1)		0 (0)
		Registered partnership or living together	8 (12)		2 (3)
	**Housing, n (%)**				
		With partner and children	65 (96)		62 (95)
		With partner	3 (4)		3 (5)
	Parent with a chronic disease, n (%)		4 (6)		7 (11)
	Job, n (%)		59 (87)		57 (88)
	Nanny, n (%)		24 (35)		35 (54)
	Experienced stress in last 6 months, n (%)		33 (49)		30 (46)
**Children**			**N=45**		**N=43**
	**Gender, n (%)**				
		Girls	22 (49)		15 (35)
		Boys	23 (51)		28 (65)
	Child’s age in years, mean (SD)		11.1 (4.6)		8.8 (5.2)
	**CKD^b^ stage child, n (%)**				
		CKD stage I (Hereditary CKD)	11 (24)		10 (23)
		CKD stage I (Nephrotic syndrome)	7 (16)		5 (12)
		CKD stage II-IV	9 (20)		9 (21)
		CKD stage V (using dialysis)	2 (4)		3 (7)
		After transplantation	16 (36)		16 (37)
	Child also under treatment at other centers, n (%)		24 (53)		21 (49)

^a^One participant did not answer the question.

^b^CKD: chronic kidney disease.

**Table 3:** Results of the five outcomes.

Outcome measure and group^a^			T0^b^, mean (SD)	T1^c^, mean (SD)	T2^d^, mean (SD)
**Child’s quality of life (CVS^e^, range scale 0-24)**					
	Group 1		7.4 (4.1)	6.8 (4.4)	5.8 (3.7)
	Group 2		7.5 (4.1)	7.1 (4.5)	6.8 (4.8)
**Parental stress (PIP^f^, range scale 42-210)**					
	**Frequency**				
		Group 1	99.0 (22.5)	95.7 (24.1)	89.3 (16.6)
		Group 2	99.7 (26.3)	97.9 (26.5)	98.0 (21.6)
	**Difficulty**				
		Group 1	87.9 (27.2)	85.3 (31.0)	74.6 (21.6)
		Group 2	80.8 (25.0)	79.0 (24.6)	78.5 (18.0)
**Parental fatigue (MFI^g^, range domain scale 4-20)**					
	**General fatigue**				
		Group 1	11.7 (3.4)	11.5 (3.8)	10.4 (3.1)
		Group 2	12.0 (3.8)	11.4 (3.9)	10.8 (4.6)
	**Physical fatigue**				
		Group 1	9.7 (2.9)	10.1 (3.4)	9.1 (2.7)
		Group 2	9.8 (3.5)	9.7 (3.5)	9.7 (3.8)
	**Mental fatigue**				
		Group 1	9.6 (3.0)	9.8 (3.4)	9.0 (3.0)
		Group 2	9.9 (3.7)	10.1 (3.8)	9.1 (3.9)
	**Reduction in motivation**				
		Group 1	9.5 (2.7)	9.3 (2.8)	8.5 (2.4)
		Group 2	9.3 (3.0)	8.9 (2.8)	9.2 (2.9)
	**Reduction in activity**				
		Group 1	9.4 (2.7)	9.5 (3.4)	8.7 (2.9)
		Group 2	9.0 (3.3)	9.2 (3.4)	8.4 (3.5)
**Self-efficacy in communication with health care professionals (PEPPI-5^h^, range scale 5-25)**					
	Group 1		21.3 (2.7)	21.1 (2.2)	21.7 (2.5)
	Group 2		21.7 (2.6)	21.8 (2.8)	20.5 (2.9)
**Family management (FaMM^i^)**					
	**Child’s daily life (range 5-25)**				
		Group 1	17.2 (4.1)	17.4 (4.2)	17.3 (4.2)
		Group 2	17.5 (4.4)	18.1 (3.8)	17.7 (4.4)
	**Condition management ability (range 12-60)**				
		Group 1	42.6 (4.3)	42.2 (3.7)	44.0 (3.0)
		Group 2	42.6 (4.5)	43.2 (4.4)	41.7 (5.0)
	**Condition management effort (range 4-20)**				
		Group 1	13.2 (3.0)	12.2 (3.0)	12.3 (3.1)
		Group 2	12.6 (3.7)	11.7 (3.9)	11.9 (3.4)
	**Family life difficulty (range 14-70)**				
		Group 1	35.2 (9.1)	35.3 (9.1)	32.4 (8.1)
		Group 2	34.3 (10.1)	33.5 (10.5)	33.5 (2.1)
	**Parental mutuality (range 8-40)**				
		Group 1	32.2 (4.4)	31.9 (4.0)	31.7 (4.1)
		Group 2	32.8 (4.0)	32.0 (4.2)	34.5 (4.8)
	**View on condition impact (range 10-50)**				
		Group 1	28.4 (4.1)	28.0 (4.5)	27.4 (4.1)
		Group 2	27.0 (5.9)	25.9 (5.7)	27.1 (4.2)

^a^Group 1: access to *e-Powered Parents* after T0; Group 2: access to *e-Powered Parents* after T1.

^b^Group 1: n=68; Group 2: n=65.

^c^Group 1: n=35; Group 2: n=51.

^d^Group 1: n=31; Group 2: n=20.

^e^CVS: Child Vulnerability Scale.

^f^PIP: Pediatric Inventory for Parents.

^g^MFI: Multidimensional Fatigue Inventory.

^h^PEPPI-5: Perceived Efficacy in Patient-Physician Interactions.

^i^FaMM: Family Management Measure.

### Process Evaluation

#### Recruitment

In the Methods section, we have described the recruitment of parents for the trial. However, recruitment also included the procedures used to maintain parents’ involvement in *e-Powered Parents* (group 1 between T0 and T1 and group 2 after T1). Parents who did not log in to the *e-Powered Parents* received email reminders, including log-in codes and a manual. Furthermore, we regularly posted newsletters on *e-Powered Parents*; parents, who had logged in once, received this newsletter via email.

After group 2 gained access to *e-Powered Parents* as well and *e-Powered Parents* became part of the daily care, JK and MK regularly discussed the program with the parents at the outpatient clinic. Meanwhile, leaflets for parents about *e-Powered Parents* were distributed during the outpatient visits. Apart from the parents, JK also informed the health care professionals involved in the daily care of children with CKD at the Pediatric Nephrology Unit about the program’s progress in their weekly multidisciplinary meetings.

#### Reach

In this study, 133 parents of 89 families participated (response rate, 44.3%), including 31 parents of children with hereditary kidney disease, 17 with nephrotic syndrome, 32 with chronic kidney failure, 9 undergoing dialysis, and 44 with renal transplantation. The community was used by 84% (57/68) of the parents in group 1 (T0-T2) and by 49% of the parents in group 2 (32/65; T1-T2; based on the IP address).

#### The Dose Received: Exposure

We separately described the dose received for the community and training platform. The majority of parents using the community logged in only once or twice; 22 parents logged in more than 51 times. Although parents in group 2 were also given access to *e-Powered Parents*, the program was most often used between T0 andT1.

**Figure 3:**
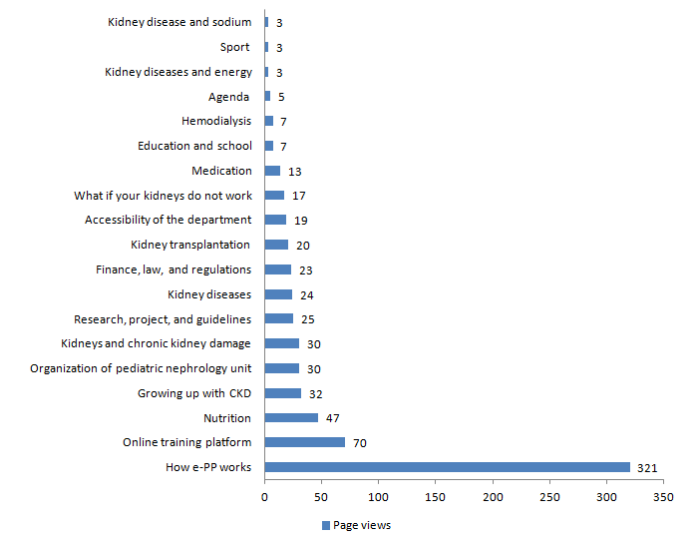
Most widely read information topics on the community between January 2015 and May 2016. CKD: chronic kidney disease; e-PP: e-Powered Parents.

The majority of parents visited the information pages on the community. Favorite topics were how the community and the training platform worked (321 and 70 page views, respectively), information about nutrition (47 page views), growing up with CKD (32 page views), and kidney diseases (30 page views; see Figure 3). Specific peritoneal dialysis topics (such as infections), recipes for public holidays, potassium and phosphate binders, and medication for blood pressure were not read at all.

On the interactive part of the community, the most widely read forum messages were about medication (98 views), kidney diseases (95 views), and transplantation (53 views). Parents responded most on topics regarding prednisone use (18 messages) and experiences with school (6 messages). The chat page was visited 109 times, although no one used it. One father wrote a blog about his child’s kidney transplantation, which was read 17 times, and 19 conversations took place through private messages. The training platform was used by 64% (85/133) of the parents; 31% (26/85) parents actually followed one or more training modules and 96% logged in only once.

**Figure 4:**
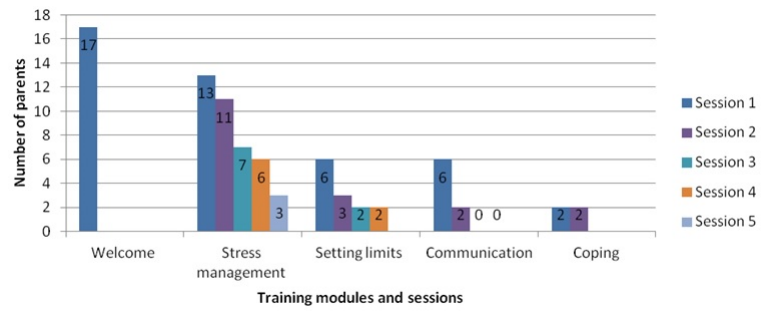
Use of training modules between January 2015 and May 2016.

Parents mainly visited the welcome module (n=17), followed by the training module stress management (Figure 4). In 3 of 4 training modules, a decrease was noted in the number of parents per session. Only the parents who followed the training module coping completed the whole training. The third and fourth sessions of the training communication were not visited at all.

The reasons given by parents for not using the community and training platform included no need (because of the stable condition of their child), other priorities, no need for support, or lack of time. Parents also mentioned that their partner was already using the program, or they mentioned a lack of knowledge on how to use the program, which led to difficulties in logging in and setting up an account.

#### The Dose Received: Satisfaction

The parents were satisfied with *e-Powered Parents*; they found it to be easy to use (30/36, 83%) and providing relevant information on the information pages and newsletters (31/36, 86% and 31/37, 84%, respectively), which were easy to read (34/36, 94% and 37/37, 100%, respectively). However, some parents did mention that the amount of information was limited (13/36, 36%) or not relevant (5/36, 14%).

The interaction part was underlined by parents as well. However, only a minority of the parents used the Web-based interaction possibilities. Some parents mentioned in the questionnaire that they do not need peer support (because they already use Facebook, among other reasons). Other parents described that the number of parents participating was limited and that the none response on questions raised was not an incentive to ask further questions.

Parents who did not use the training platform indicated that the modules did not correspond with their request for support. One barrier mentioned by parents was the extra log-in for the Web-based training platform. However, parents who did use the modules were satisfied; they found the instructions to be clear and the modules easy to use.

#### Context

In the focus group interview, health care professionals indicated that they found it important to keep up with (Web-based) developments. They described *e-Powered Parents* as essential because it provides parents with accessible, reliable, and objective information, which is usually difficult to find. However, the implementation and use of *e-Powered Parents* in daily care (such as by referring parents during their consultation) needs more attention because *“It is not a routine yet.”* The extra log-in for parents to use the Web-based training platform was not desirable either. Health care professionals put particular stress on their responsibility for the content of the information, although they also mentioned that it is “*not our core business*,” hence, their worry about the continuity of *e-Powered Parents* in the future.

## Discussion

### Principal Findings

This is the first effect study and process evaluation of an online support program for parents of children with stage I-V CKD. In this feasibility study, the first objective was to analyze the outcome measures most likely to capture the potential benefit. The chosen outcome measures were likely to capture the potential effect, except for self-efficacy in communication with health care professionals, measured by the PEPPI-5 scale; this scale shows ceiling effects and high percentages of parents showing no change between the measurement times. This skewed distribution, meaning that the majority of the parents were already confident in asking questions and discussing their problems with health care professionals, could possibly be explained by the long-standing relationship of the parents with the health care professionals and the small team of health care professionals in pediatric nephrology.

The second objective was to evaluate the potential effectiveness and effect size. Although the parents and health care professionals were enthusiastic about *e-Powered Parents*, no statistically significant effect was found on children’s quality of life, parental stress and fatigue, family management, or parents’ self-efficacy in communication in both intention-to-treat and per-protocol analyses.

In the process evaluation (third objective), we noticed that the majority of the parents used the community to read the information and that only a few parents actually used the training platform. This could explain the very small effects found in this study: knowledge only does not lead to behavioral changes [31]. Other determinants (such as attitude and self-efficacy) could have been influenced, although strategies to change these determinants were mainly integrated into the training platform and not into the community [14]. On the other hand, parents and health care professionals underlined the importance of evidence-based information on *e-Powered Parents*, providing parents with reliable and up-to-date information about their child’s disease and treatment options. The uncertainty about the trustworthiness of the information that parents find on internet is an often-heard reason among parents for not using Web-based information resources [32-35]. However, they do express a need for reliable information to manage their uncertainty, make decisions regarding their child’s treatment, and stimulate the dialogue with the care providers of their child [32-34]. The minimal use of the interaction part of the community and the training platform is remarkable. In order to develop this intervention, we conducted an extensive needs assessment consisting of a literature study and 5 focus group interviews with parents [13]. This needs assessment revealed that online peer support was (the most) frequently mentioned need of the parents, and it is often used by parental caregivers for emotional needs [7,8,33]. Even so, the Web-based interaction options of *e-Powered Parents* were not often used. Parents might feel reluctant to share their experiences because of the monitoring by health care professionals. Scharer [34] suggested a continuous clarification of the role of the professionals in online support groups. However, the option to send private messages on *e-Powered Parents* (which could not be monitored by the health care professionals) was not used very much either.

Another possible explanation for the minimal use of *e-Powered Parents* is the heterogeneity of the study population, consisting of fathers and mothers of children with different ages, different kidney diseases and stages (CKD I-V), and treatments. *e-Powered Parents* was not tailored, while the support needs of these heterogeneous groups differed. Swallow et al [36], who developed an online support program for parents of children with stage III-V CKD in the United Kingdom, did not find a significant effect in their feasibility study either.

### Strengths and Limitations

We believe that our feasibility study has numerous methodological strengths. By conducting process evaluation, which is recommended when evaluating complex interventions [16], we gained more insight into our recruitment procedures, how many parents we contacted, how actively they engaged, and how satisfied they were. Additionally, we gained more knowledge about the context and how this affected the implementation and use of *e-Powered Parents.* Using the framework of Linnan and Steckler [28], we were able to do this in a structured way. Additionally, using quantitative and qualitative research methods, we increased our understanding of the outcomes, enabling us to improve the intervention for the future [16].

However, some limitations need to be mentioned as well. First, the intervention *e-Powered Parents* consisted of two websites: the community (consisting of the information and interactive part) and the training platform. As described in the Methods section, each website comprised different systems to register and analyze the log-in data and could, therefore, not be interpreted in the same way. Most notably, the use of Google Analytics (to analyze the community data) was challenging because the log-in data were registered on IP addresses; parents who used *e-Powered Parents* from different locations were consequently registered as different users. Moreover, after T1, when the program became part of the daily care, we were not able to exclude parents who were not a part of the trial. Hence, the presented log-in data of the community could be an overestimation. We tried to correct this by checking the users’ account on the community website (did the parents who were part of the trial actually logged in or not?). This problem did not apply to the log-in data of the training platform because this was registered based on the email address of the parents. Parents who did not participate in the trial could easily be excluded from the analysis.

Second, a formal power calculation was not possible in this study, leading us to include as many parents as possible. Although 133 parents filled in the baseline questionnaire, only 38.3% (51/133) parents filled in the questionnaire at T2. This high lost to the follow-up rate could severely comprise the study’s validity and reduce the chances of detecting a true effect [37]. Possible explanations for this high lost to follow-up rate are the amount of questions in the questionnaires and an extra unplanned measurement at T2. We decided to conduct an extra measurement, aiming to gain insights into the long-term effects; however, the parents were not aware of this extra measurement at the start of the study.

Finally, we opted for outcomes at a child and parent level, such as quality of life, fatigue, and stress, but not at an organizational level, such as the number of outpatient clinic visits. It would be worthwhile to consider such outcomes in a full-scale RCT.

### Conclusions

Parents and health care professionals were very positive about *e-Powered Parents*, and they underlined its importance, yet no significant effect of *e-Powered Parents* was found in this study on the child’s quality of life, parental stress and fatigue, parents’ self-efficacy in communication, and family management. This could be explained by both the minimal use of *e-Powered Parents* and the heterogeneity of the participants. To continue parents’ participation, we recommend a tailored intervention based on the different CKD stages and needs of the parents. Nevertheless, further studies are necessary to determine whether and how online programs can be used to support the parents of children with CKD in the management of their child’s disease.

## Appendix

Multimedia Appendix 1 Content of e-Powered Parents.

Multimedia Appendix 2 Percentage of parents scoring floor and ceiling effectts.

Multimedia Appendix 3 Percentage of parents showing no change in score between T0, T1, and T2.

Multimedia Appendix 4 Overview of mixed-model analysis intention to treat: T0-T1 and T0-T2.

Multimedia Appendix 5 Overview of mixed-model analysis per protocol: T0-T1 and T0-T2.

Multimedia Appendix 6 CONSORT‐EHEALTH checklist (V 1.6.1).

## Abbreviations

CKD: chronic kidney disease
CVS: Child Vulnerability Scale
FaMM: Family Management Measure
IP: Internet protocol
MFI: Multidimensional Fatigue Inventory
PEPPI-5: Perceived Efficacy in Patient-Physician Interactions
PIP: Pediatric Inventory for Parents
RCT: randomized controlled trial
