# Supplementary material for: Effect and Process Evaluation of e-Powered Parents, a Web-Based Support Program for Parents of Children With a Chronic Kidney Disease: Feasibility Randomized Controlled Trial
Source: J Med Internet Res. 2018 Aug 1;20(8):e245. doi: 10.2196/jmir.9547 (PMC6094085; doi:10.2196/jmir.9547)
Supplement: Multimedia Appendix 1 [file jmir_v20i8e245_app1.pdf]

Figure 1. Content of *e-Powered Parents*

Website	Part	Topics
Community	*Information part*	• Information and instructions how *e-Powered Parents* (community and training platform) works, including an online manual
		• Organization of pediatric nephrology unit (accessibility and team members)
		• Kidneys, kidney diseases and chronic kidney damage
		• Nutrition (e.g. proteins, phosphates, sodium, potassium, water, energy and recipes)
		• Medication (e.g. blood pressure, iron deficiency, erythropoietin, vitamins, potassium binders, growth hormone, immune suppressants)
		• Hemodialysis (e.g. shunt and dialysis catheter) and peritoneal dialysis (e.g. catheter, infections, continuous ambulatory peritoneal dialysis)
		• Kidney transplantation (e.g. blood types and compatibility, rejection of transplant, living donor, screening and preparation, during and after)
		• Growing up with chronic kidney disease (e.g. transition from a pediatric nephrologist to an adult nephrologist)
		• Education, school, and children's hospital school
		• Finance, laws, and regulations
		• Sport
		• Scientific research, projects, and guidelines
	Interaction part	• Blog
		• Forum (with items such as nutrition, medication, dialysis, transplantation, kidney diseases, school and education, holidays, finance and insurance)
		• Chat
		• Private messages
Training platform	*Training modules*	• Welcome
		Stress management module<br>• Welcome<br>• Symptoms of stress<br>• Coping with stress<br>• Creating a personal stress management plan
		Setting limits module<br>• Welcome<br>• Why saying no is important<br>• How to say no<br>• Handing over care
		Communication module<br>• Welcome<br>• Active listening<br>• Asking questions<br>• Being assertive<br>• Communication in practice (healthcare professional, child, and other children in family)
		Coping with your child's CKD module<br>• Coping<br>• Tips that could help you coping with your grief
