# Supplementary material for: Effect and Process Evaluation of e-Powered Parents, a Web-Based Support Program for Parents of Children With a Chronic Kidney Disease: Feasibility Randomized Controlled Trial
Source: J Med Internet Res. 2018 Aug 1;20(8):e245. doi: 10.2196/jmir.9547 (PMC6094085; doi:10.2196/jmir.9547)
Supplement: Multimedia Appendix 2 [file jmir_v20i8e245_app2.pdf]

Scale	Subscale	Floor or ceiling effects	TO	TO	T1	T1	T2	T2
			**Group1**	**Group 2**	**Group 1**	**Group 2**	**Group 1**	**Group 2**
**CVS**		Floor (0); n (%)	2 (2,8)	1 (1,5)	1 (2,6)	3 (5,8)	1 (3,1)	2 (10)
		Ceiling (24); n (%)	0 (0)	0 (0)	0 (0)	0 (0)	0 (0)	0 (0)
**PIP**	Total score Frequency	Floor (42); n (%)	0 (0)	0 (0)	0 (0)	1 (1,9)	0 (0)	0 (0)
		Ceiling (210); n (%)	0 (0)	0 (0)	0 (0)	0 (0)	0 (0)	0 (0)
	Total score Difficulty	Floor (42); n (%)	0 (0)	1 (1,5)	0 (0)	2 (3,8)	1 (3,1)	0 (0)
		Ceiling (210); n (%)	0 (0)	0 (0)	0 (0)	0 (0)	0 (0)	0 (0)
**MFI**	General fatigue	Floor (4); n (%)	1 (1,5)	1 (1,5)	1 (2,9)	1 (2,0)	1 (3,2)	2 (10)
		Ceiling (20); n (%)	0 (0)	1 (1,5)	0 (0)	0 (0)	0 (0)	1 (5)
	Physical fatigue	Floor (4); n (%)	3 (4,4)	4 (6,2)	2 (5,7)	3 (5,9)	2 (6,5)	3 (15)
		Ceiling (20); n (%)	0 (0)	0 (0)	0 (0)	0 (0)	0 (0)	0 (0)
	Mental fatigue	Floor (4); n (%)	4 (5,9)	5 (7,7)	2 (5,7)	2 (3,9)	2 (6,5)	2 (10)
		Ceiling (20); n (%)	0 (0)	0 (0)	0 (0)	1 (2,0)	0 (0)	0 (0)
	Reduction in motivation	Floor (4); n (%)	1 (1,5)	3 (4,6)	2 (5,7)	3 (5,9)	1(3,2)	2 (10)
		Ceiling (20); n (%)	0 (0)	0 (0)	0 (0)	0 (0)	0 (0)	0 (0)
	Reduction in activity	Floor (4); n (%)	3 (4,4)	6 (9,2)	3 (8,6)	1 (2,0)	3 (9,7)	3 (15)
		Ceiling (20); n (%)	0 (0)	0 (0)	0 (0)	1 (2,0)	0 (0)	0 (0)
**Peppi**		Floor (5); n (%)	0 (0)	0 (0)	0 (0)	0 (0)	0 (0)	0 (0)
		Ceiling (25); n (%)	13 (19,1)	14 (21,5)	5 (14,3)	14 (27,5)	6 (19,4)	5 (25)
**FaMM**	Child's daily care	Floor (5); n (%)	0 (0)	0 (0)	0 (0)	0 (0)	0 (0)	0 (0)
		Ceiling (25); n (%)	3 (4,2)	3 (4,5)	3 (7,9)	2 (3,8)	1 (3,1)	2 (10)
	Condition management ability	Floor (12); n (%)	0 (0)	0 (0)	0 (0)	0 (0)	0 (0)	0 (0)
		Ceiling (60); n (%)	0 (0)	0 (0)	0 (0)	0 (0)	0 (0)	0 (0)
	Condition management effort	Floor (4); n (%)	0 (0)	1 (1,5)	0 (0)	1 (1,9)	0 (0)	0 (0)
		Ceiling (20); n (%)	0 (0)	1 (1,5)	0 (0)	2 (3,8)	1 (3,1)	0 (0)
	Family life difficulty	Floor (14); n (%)	0 (0)	1 (1,5)	0 (0)	2 (3,8)	0 (0)	0 (0)
		Ceiling (70); n (%)	0 (0)	0 (0)	0 (0)	0 (0)	0 (0)	0 (0)
	View on condition impact	Floor (10); n (%)	0 (0)	0 (0)	0 (0)	0 (0)	0 (0)	0 (0)
		Ceiling (50); n (%)	0 (0)	0 (0)	0 (0)	0 (0)	0 (0)	0 (0)
	Parental mutuality	Floor (8); n (%)	0 (0)	0 (0)	0 (0)	0 (0)	0 (0)	0 (0)
		Ceiling (40); n (%)	1 (1,5)	2 (3,0)	1 (2,6)	2 (3,8)	2 (6,3)	0 (0)
