# Supplementary material for: Effect and Process Evaluation of e-Powered Parents, a Web-Based Support Program for Parents of Children With a Chronic Kidney Disease: Feasibility Randomized Controlled Trial
Source: J Med Internet Res. 2018 Aug 1;20(8):e245. doi: 10.2196/jmir.9547 (PMC6094085; doi:10.2196/jmir.9547)
Supplement: Multimedia Appendix 3 [file jmir_v20i8e245_app3.pdf]

Scale	Subscale	Time of measurement	Group 1 (%)	Group 2 (%)	Total (%)
CVS		T0-T1	9.7	10.6	10.1
		T0-T2	4.2	9.1	6.5
PIP	Total score frequency	T0-T1	2.8	3.0	2.9
		T0-T2	2.8	1.5	2.2
	Total score difficulty	T0-T1	1.4	1.5	1.4
		T0-T2	0.0	0.0	0.0
MFI	General fatigue	T0-T1	6.9	12.1	9.4
		T0-T2	11.1	7.6	9.4
	Physical fatigue	T0-T1	8.3	9.1	8.7
		T0-T2	8.3	4.5	6.5
	Mental fatigue	T0-T1	12.5	9.1	10.9
		T0-T2	12.5	7.6	10.1
	Reduction in motivation	T0-T1	13.9	16.7	15.2
		T0-T2	11.1	4.5	8.0
	Reduction in activity	T0-T1	12.5	6.1	9.4
		T0-T2	8.3	4.5	6.5
PEPPI		T0-T1	33.3	48.5	40.6
		T0-T2	30.6	19.7	25.4
FaMM	Child’s daily care	T0-T1	9.7	16.7	13.0
		T0-T2	5.6	4.5	5.1
	Condition management ability	T0-T1	5.6	12.1	8.7
		T0-T2	5.6	4.5	5.1
	Condition management effort	T0-T1	4.2	10.6	7.2
		T0-T2	8.3	7.6	8.0
	Family life difficulty	T0-T1	2.8	12.1	7.2
		T0-T2	2.8	1.5	2.2
	View on condition impact	T0-T1	6.9	9.1	8.0
		T0-T2	1.4	3.0	2.2
	Parental mutuality	T0-T1	12.5	9.1	10.9
		T0-T2	5.6	7.6	6.5
