# Supplementary material for: Effect and Process Evaluation of e-Powered Parents, a Web-Based Support Program for Parents of Children With a Chronic Kidney Disease: Feasibility Randomized Controlled Trial
Source: J Med Internet Res. 2018 Aug 1;20(8):e245. doi: 10.2196/jmir.9547 (PMC6094085; doi:10.2196/jmir.9547)
Supplement: Multimedia Appendix 4 [file jmir_v20i8e245_app4.pdf]

		Intention to treat T0-T1			Intention to treat T0-T2		
**Scale**	**Subscales**	**SES[1]**	**95% CI[2]**	**p-Value**	**SES[1]**	**95% CI[2]**	**p-Value**
CVS		-.19	-2.16 to 0.54	.24	-.21	-2.13 to 0.39	.17
PIP	Frequency	-.22	-13.51 to 3.07	.22	-.15	-11.71 to 4.36	.37
	Difficulty	.00	-9.68 to 9.42	.98	.07	-7.15 to 10.80	.69
MFI	General fatigue	.02	-0.94 to 1.10	.88	-.03	-1.14 to 0.95	.86
	Physical fatigue	.12	-0.61 to 1.38	.44	.08	-0.74 to1.25	.61
	Mental fatigue	-.11	-1.60 to 0.85	.55	-.15	-1.63 to 0.61	.37
	Reduced motivation	.05	-0.90 to 1.20	.78	.04	-0.90 to 1.10	.85
	Reduced activity	-.02	-1.09 to 1.00	.93	-.08	-1.24 to 0.76	.64
PEPPI		-.11	-1.20 to 0.60	.51	-.06	-1.05 to 0.74	.74
FaMM	Child's daily life	-.07	-1.57 to 0.95	.63	-.02	-1.29 to 1.10	.88
	Condition management ability	-.19	-2.23 to 0.66	.29	-.16	-2.14 to 0.82	.38
	Condition management effort	-.02	-1.18 to 1.02	.89	-.10	-1.35 to 0.64	.48
	Family life difficulty	-.03	-3.00 to 2.37	.82	-.02	-2.96 to 2.21	.78
	Parental mutuality	.15	-0.88 to 2.11	.42	.12	-1.01 to 1.99	.52
	View on condition impact	-.01	-1.62 to1.57	.97	-.05	-1.76 to1.25	.74

[1]SES: Standardized effect size, [2]CI: Confidence Interval
