# Supplementary material for: Effect and Process Evaluation of e-Powered Parents, a Web-Based Support Program for Parents of Children With a Chronic Kidney Disease: Feasibility Randomized Controlled Trial
Source: J Med Internet Res. 2018 Aug 1;20(8):e245. doi: 10.2196/jmir.9547 (PMC6094085; doi:10.2196/jmir.9547)
Supplement: Multimedia Appendix 5 [file jmir_v20i8e245_app5.pdf]

		Per protocol T0-T1			Per protocol T0-T2		
**Scale**	**Subscales**	**SES[1]**	**95% CI[2]**	**p-Value**	**SES[1]**	**95% CI[2]**	**p-Value**
CVS		-.19	-2.17 to 0.54	.24	-.20	-2.10 to 0.41	.19
PIP	Frequency	-.01	-13.81 to 2.81	.19	-.17	-12.06 to 4.03	.33
	Difficulty	-.23	-9.75 to 9.43	.97	.07	-7.12 to 10.89	.68
MFI	General fatigue	.02	-0.97 to 1.08	.91	-.03	-1.16 to 0.94	.83
	Physical fatigue	.10	-0.67 to 1.33	.52	.06	-0.79 to 1.20	.69
	Mental fatigue	-.12	-1.65 to 0.82	.51	-.16	-1.67 to 0.58	.34
	Reduced motivation	.05	-0.91 to 1.20	.79	.04	-0.91 to 1.10	.85
	Reduced activity	-.03	-1.14 to 0.96	.87	-.09	-1.27 to 0.73	.59
PEPPI		-.13	-1.23 to 0.57	.47	-.07	-1.09 to 0.71	.68
FaMM	Child’s daily life	-.06	-1.51 to 1.01	.70	-.01	-1.25 to 1.15	.94
	Condition management ability	-.17	-2.17 to 0.73	.33	-.14	-2.05 to 0.89	.44
	Condition management effort	-.03	-1.22 to 0.99	.83	-.11	-1.38 to 0.62	.46
	Family life difficulty	-.03	-2.95 to 2.39	.84	-.04	-2.91 to 2.24	.80
	Parental mutuality	.15	-0.86 to 2.13	.40	.12	-1.00 to 2.00	.51
	View on condition impact	-.01	-1.63 to 1.57	.97	-.05	-1.76 to 1.25	.74

[1]SES: Standardized effect size, [2]CI: Confidence Interval
